# Characterization of gut microbiome in mice model of depression with divergent response to escitalopram treatment

**DOI:** 10.1038/s41398-021-01428-1

**Published:** 2021-05-20

**Authors:** Jiajia Duan, Yu Huang, Xunmin Tan, Tingjia Chai, Jing Wu, Hanping Zhang, Yifan Li, Xi Hu, Peng Zheng, Ping Ji, Libo Zhao, Deyu Yang, Liang Fang, Jinlin Song, Peng Xie

**Affiliations:** 1 NHC Key Laboratory of Diagnosis and Treatment on Brain Functional Diseases, Chongqing Medical University, Chongqing, China; 2 The M.O.E. Key Laboratory of Laboratory Medical Diagnostics, the College of Laboratory Medicine, Chongqing Medical University, Chongqing, China; 3 Department of Neurology, The First Affiliated Hospital of Chongqing Medical University, Chongqing, China; 4 College of Biomedical Engineering, Chongqing Medical University, Chongqing, China; 5 College of Stomatology, Chongqing Medical University, Chongqing, China; 6 Chongqing Key Laboratory for Oral Diseases and Biomedical Sciences, Chongqing, China; 7 Department of Neurology, Yongchuan Hospital of Chongqing Medical University, Chongqing, China; 8 Key Laboratory of Psychoseomadsy, Stomatological Hospital of Chongqing Medical University, Chongqing, China

**Keywords:** Depression, Molecular neuroscience

## Abstract

Depression is a common and heterogeneous mental disorder. Although several antidepressants are available to treat the patients with depression, the factors which could affect and predict the treatment response remain unclear. Here, we characterize the longitudinal changes of microbial composition and function during escitalopram treatment in chronic unpredictable mild stress (CUMS) mice model of depression based on 16 S rRNA sequencing and metabolomics. Consequently, we found that escitalopram (ESC) administration serves to increase the alpha-diversity of the gut microbiome in ESC treatment group. The microbial signatures between responder (R) and non-responder (NR) groups were significantly different. The R group was mainly characterized by increased relative abundances of genus *Prevotellaceae_UCG-003*, and depleted families *Ruminococcaceae* and *Lactobacillaceae* relative to NR group. Moreover, we identified 15 serum metabolites responsible for discriminating R and NR group. Those differential metabolites were mainly involved in phospholipid metabolism. Significantly, the bacterial OTUs belonging to family *Lachnospiraceae*, *Helicobacteraceae,* and *Muribaculaceae* formed strong co-occurring relationships with serum metabolites, indicating alternations of gut microbiome and metabolites as potential mediators in efficiency of ESC treatment. Together, our study demonstrated that the alterations of microbial compositions and metabolic functions might be relevant to the different response to ESC, which shed new light in uncovering the mechanisms of differences in efficacy of antidepressants.

## Introduction

Depression, a debilitating mental disorder^1,2^, has detrimental impact on health systems and psychological well-being. Treatment options for depression include psychotherapy and antidepressants such as selective serotonin reuptake inhibitors (SSRIs) and tricyclic antidepressants (TCAs)^3^. Escitalopram (ESC) is widely used for treatment of depression, which is a highly selective inhibitor of the serotonin transporter protein and characterized by rapid onset of antidepressant activity^4^. In the clinical practice, the currently available antidepressants lack efficacy for some patients with depression^5,6^, the response and remission rates in depressive patients after ESC monotherapy were 68% and 46%, respectively^7^. In patients who have previously responded to treatment, they are likely to develop drug-resistant over time^8^. However, it’s barely known about what distinguishes between patients responding to treatment or not, and the factors affecting and predicting the ESC treatment response are still unclear. Thus, it is crucial to investigate the factors that associated with the antidepressant efficacy.

In recent years, it was reported that the disturbances of gut microbial ecosystem were linked with psychological conditions, including bipolar disorder, autism spectrum disorder, schizophrenia, and depression^9–13^. Patients with those diseases show distinct compositional or functional changes in their gut microbiome. For example, our previous studies showed that major depressive disorder (MDD) patients were characterized by disturbance of gut microbiome^10,14^. Fecal microbiota transplantation (FMT) of germfree mice with “depression microbiota” derived from MDD patients could result in depression-like behaviors^14^. Moreover, we found that the gut microbiome might be involved in the onset of depressive-like behaviors through modulating the central and peripheral glycerophospholipid metabolism^11^. Meanwhile, a recent study showed that many commonly used non-antibiotic drugs enabled to modulate the microbial composition or function^15,16^. For example, ketamine, a novel and rapid-acting antidepressant, was able to increase the relative abundance of *Lactobacillus*, *Turicibacter,* and *Sarcina*^17^. The antidepressant effects of ketamine in CSDS model were reported partly mediated by the restoration of the gut microbiota^18^. In contrast, gut microbiome could also affect an individual’s response to a specific drug directly by chemical modification and altering the bioactivity, bioavailability, or toxicity of drugs^19,20^. For examples, gut microbiota containing tyrosine decarboxylase (TDC) can convert levodopa to dopamine, thus affecting the efficacy in the treatment of Parkinson’s disease^21^. Moreover, gut microbiome was shown to improve the efficacy of Programmed Death-1 (PD-1) and Programmed Death Ligand-1 (PD-L1) blockers in different cancer patients^22^. A research shown that 176 of 271 drugs (66%), including antipsychotics, were chemically modified by at least one kind of bacterial stain^23^. However, data concerning the impact of the gut microbiome on response to antidepressant in depressive individuals have not been reported. Thus, it is interesting to explore how gut microbiome associated with the different antidepressant response.

In present study, CUMS mice model was constructed to mimics the stressors suffered by human beings^24^. After the initial 4 weeks of CUMS, the animals were administered either ESC or vehicle for another 4 weeks. Animals treated with ESC were divided into ESC responders and ESC non-responders by behavioral results. Based on 16 S rRNA sequencing and metabolomics, we sought to investigate the characteristics of gut microbiome that associated with the ESC treatment responses in the CUMS model, to provide new evidences to understand how gut microbiome impacts drug effectiveness in depression.

## Materials and methods

### Animal experiments

Male adult C57BL/6 mice (4–6 weeks of age) were used for establishing CUMS model of depression. Animals were housed under relative steady conditions (12 h light-dark cycle at a humidity of 55 ± 5% and constant temperature of 23 ± 2 °C) and allowed food and water ad libitum. The control mice were housed together, while the CUMS-treated mice were housed individually. All animals were purchased from the experimental animal center of Chongqing Medical University (Chongqing, China). Ethics approval was obtained from the Ethics Committee of Chongqing Medical University (Approval No. 20160331). Care and treatment of animals were in accordance with the requirements of the National Institutes of Health Guidelines for Animal Research.

### Experimental design in CUMS procedure

All animals were randomly allocated into groups. With the exception of those in the control group, all animals were subjected to the mild stress protocol in an unpredictable manner for 8 weeks (Fig. S1). The protocol consisted of seven stressors: water deprivation for 24 h, food deprivation for 24 h, restraint stress for 3 h, overnight illumination for 12 h, crowding mice into an empty bottle (6 h), stroboscopic lighting (12 h), and a soiled cage environment (500 mL water added to 250 g sawdust bedding) for 24 h. After the initial 4 weeks of exposure to mild stress, the animals were administered either ESC or vehicle. Escitalopram oxalate (ESC, Sigma–Aldrich, Saint Louis, USA) was dissolved in distilled water and administered by oral gavage at a dose of 10 mg/kg^25,26^. Oral gavage was performed by using a reusable, straight, 7-gauge stainless steel feeding needle. Drug or vehicle was administrated once daily for 4 weeks at 8:00 AM. Stress was continued during the entire period of treatment, and the animals treated with ESC were segregated into drug responders and drug non-responders.

### Behavioral assays

Behavioral analysis was conducted by individuals blinded to the experimental conditions. The experiments were carried out in a soundproof room. All the animals’ behavior was recorded and measured using video tracking software.

The steps for sucrose preference test are given below. Mice were single housed and habituated to 1% sucrose solution and water for 2 days without any stress. The positions of the bottles were switched at the 24th hour. The mice were then deprived of food and water for 12 h. The mice were exposed to two bottles in the dark phase for 12 h, one containing with 1% sucrose solution and the other with tap water. Total consumption of each fluid was measured and sucrose preference ratio was calculated. The definition of an ESC responder was a minimum 10% increase in sucrose intake compared to anhedonic level^27,28^.

The steps for forced swim test are given below. The mice were placed individually in a Plexiglas cylinder (30 cm in height and 15 cm in diameter), filled with 15 cm of water (24 ± 1 °C). Immobility was defined as the absence of all motion with the exception of movements required to keep the mouse’s head above water.

### 16 S rRNA gene sequencing and data processing

Fecal samples from mice were collected immediately after defecation into a sterile tube, and quickly freeze with liquid nitrogen. After that, it was stored in a refrigerator at −80 °C prior to analyses. DNA extraction and amplification of the 16 S rRNA gene were performed as previously described^13^. Briefly, DNA was extracted from stool samples using the E.Z.N.A^®^ DNA kit (Omega Bio-Tek, USA). The V3-V4 hypervariable regions of the bacterial 16 S rRNA gene were amplified with primers 338 F (5ʹ- ACTCCTACGGGAGGCAGCAG-3ʹ) and 806 R (5ʹ-GGACTACHVGGGTWTCTAAT-3ʹ) by the thermocycler PCR system (GeneAmp 9700, ABI, USA). The PCR reaction was performed by the following program: denaturation at 95 °C for 3 min, followed by 27 cycles (denaturation at 95 °C for 30 s, annealing at 55 °C for 30 s, elongation at 72 °C for 45 s), finally, extension at 72 °C for 10 min. Then the PCR reaction was conducted in triplicate in a 20 μL mixture (4 μL of 5 × FastPfu buffer, 2 μL of dNTPs, 0.8 μL of each primer, 0.4 μL of FastPfu polymerase and 10 ng of template DNA). 2% agarose gels was used to separate the PCR products, which was then purified by the AxyPrep DNA Gel Extraction Kit (Axygen Biosciences, Union City, CA, USA) and quantified using QuantiFluor™-ST (Promega, USA) according to the manufacturer’s protocol. Purified amplicons were pooled in equimolar concentrations and paired-end sequenced (2 × 300) on an Illumina MiSeq platform (Illumina, San Diego, USA) according to the standard protocols by Majorbio Bio-Pharm Technology Co. Ltd. (Shanghai, China). The raw reads were deposited into the NCBI Sequence Read Archive (SRA) database (Accession Number: SRP302512).

The raw fastq files were quality-filtered using Trimmomatic and merged by FLASH. Operational taxonomic units (OTUs) clustering was performed using UPARSE (version 7.1, http://drive5.com/uparse/) with 97% similarity cutoff. The RDP classifier algorithm (http://rdp.cme.msu.edu/) was used to analyze the taxonomy of each 16 S rRNA gene sequence against the Silva 16 S rRNA database, a confidence threshold was set at 70%.

### Gas chromatography—mass spectrometry

The details of the metabolomics based on GC-MS method were similar to our previous study^11^. Briefly, 80 μL of plasma sample was dissolved in methanol with 2-chloro-l-phenylalanine as internal standard. Subsequently, ice-cold mixture of methanol and acetonitrile was added and vortexed for 1 min and placed at −20 °C for 10 min, then ultrasonicated at ice-water and centrifuged (15,000 rpm at 4 °C for 10 min). 100 μL of supernatant was dried, and 80 μL of methoxylamine hydrochloride in pyridine was subsequently added, and vortexed vigorously for 2 min and incubated at 37 °C for 90 min. BSTFA and n-hexane was added into the mixture, and then derivatized at 70 °C for 60 min. The samples were placed at ambient temperature for 30 min before GC-MS analysis. The derivatized samples were analyzed on an Agilent 7890 A gas chromatography system coupled to an Agilent 5975 C MSD system (Agilent, CA). The temperature of MS quadrupole, and ion source (electron impact) was set to 150, and 230 °C, respectively. Mass data was acquired in a full-scan mode (m / z 50-600), and the solvent delay time was set to 5 min. The acquired MS data from GC − MS were analyzed by ChromaTOF software (v 4.34, LECO, St Joseph, MI). And Metabolites were qualitatived by the Fiehn database, which is linked to the ChromaTOF software. The resulting data were normalized to the total peak area of each sample and imported into a SIMCA (version 14.0, Umetrics, Umeå, Sweden), the orthogonal partial least squares-discriminant analysis (OPLS-DA) were performed to identify the discriminant metabolites with the significance threshold of variable importance plot (VIP)å 1.0 and *P* < 0.05, OPLS-DA models were also validated by a permutation analysis (200 times).

### Statistical analysis

The α-diversity indexes were assessed according to richness (Ace and Chao) and diversity (Shannon and Simpson). Beta-diversity was assessed with package “vegan” in R (version R-3.3.1), and generated on the basis of Principal co-ordinates analysis (PCoA). Descriptive modelling and discriminative variable selection were evaluated by partial least squares-discriminant analysis (PLS-DA). ANOSIM tests was performed to identify differences in β-diversity among groups. The key bacterial taxa and metabolites responsible for discrimination among the groups, were identified using linear discriminant analysis effective size (LEfSe; http://huttenhower.sph.harvard.edu/galaxy/root?tool_id=lefse_upload). Only LDA > 2.0 at a *P* < 0.05 were considered significantly enriched.

Multiple group comparisons were assessed using one-way analysis of variance (ANOVA) followed by LSD as a post-hoc test, and the two-tailed Student’s *t* test or Wilcoxon rank-sum test were performed to determine differences between each set of two groups. The correlation between microbial taxa with behavioral tests and metabolites was tested by Spearman correlation analyses. Statistical analyses were conducted using the software SPSS, R package, and plots were generated from R and GraphPad Prism version 8.0.

## Results

### Behavioral assessment

**Fig. 1:**
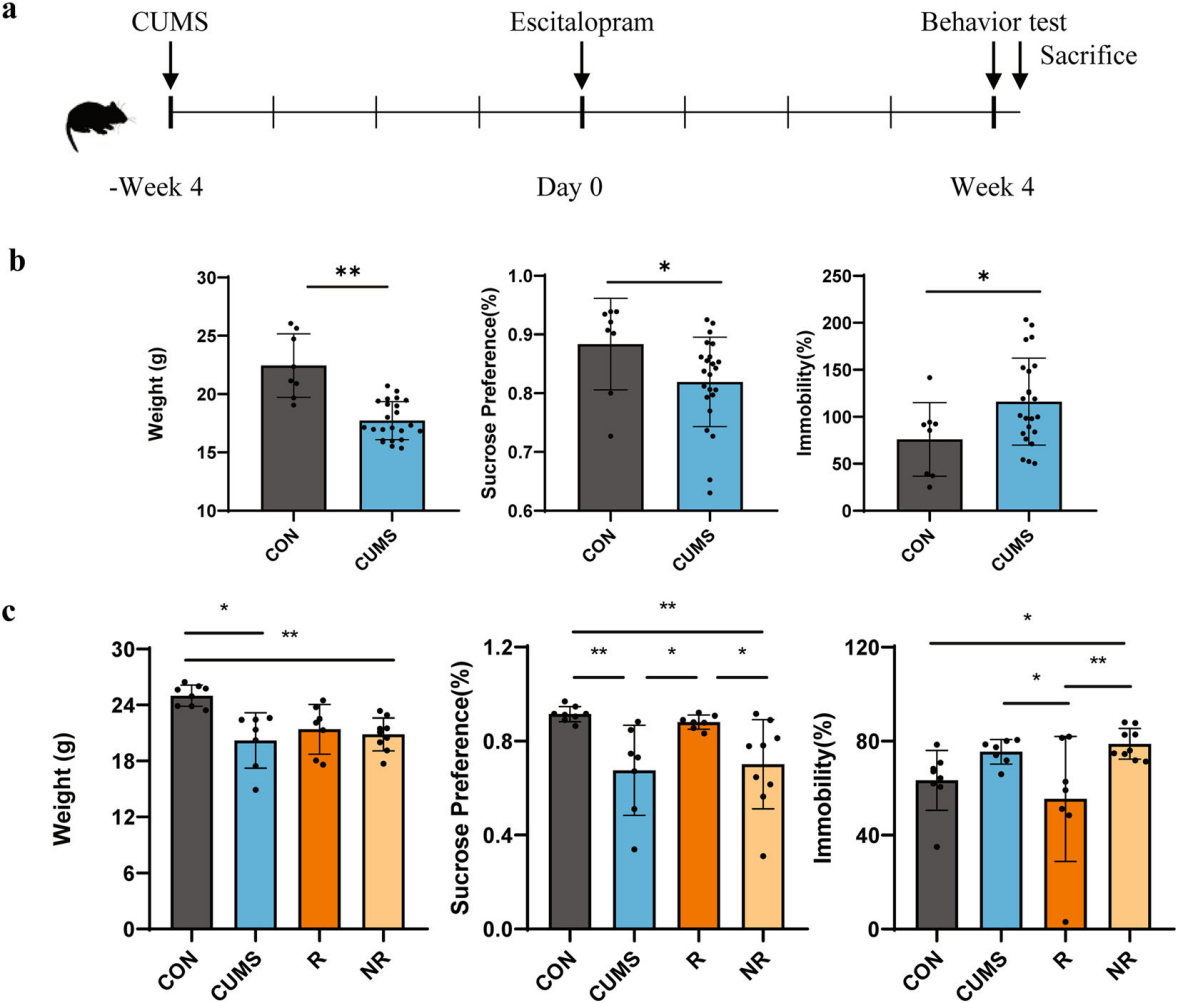
Schematic of experimental protocols and procedures and behavioral results. **a** Flow diagram of experiment. Analysis of weight, sucrose preference in SPT and immobility (%) in FST of the mice after 4 weeks of CUMS (**b**) and 4 weeks of escitalopram administration (**c**). (*p < 0.05, **p < 0.01, One-way analysis of variance). FST, forced swim test. SPT, sucrose preference test.

The experimental scheme was sketched in Fig. 1a, the depressive-like behaviors in mice were evaluated by the sucrose preference test (SPT) and forced swimming test (FST). After 4 weeks of CUMS exposure, significant decreases of sucrose drinking in the SPT (R: 0.884 ± 0.028 [mean ± SEM]; NR: 0.819 ± 0.016; *p* = 0.049) and increases of immobility time in FST (R: 75.883 ± 13.835 [mean ± SD]; NR: 116.135 ± 9.660; *p* = 0.036) were observed in the CUMS group compare to control group (Fig. 1b), which indicated the development of depressive-like behaviors in mice. After the CUMS protocol, the animals were administered either ESC or vehicle, and were divided into four groups: control (CON, *N* = 8), CUMS with vehicle (CUMS, *N* = 7), responders to ESC treatment (R, *N* = 7), non-responders to ESC treatment (NR, *N* = 9). The definition of an ESC responder was a minimum 10% increase in sucrose intake compared to anhedonic level^27,28^. As shown in Fig. 1c, compared to R group, the NR group had a lower sucrose preference (R: 0.881 ± 0.011 [mean ± SEM]; NR: 0.702 ± 0.063; *p* = 0.016) and an increased immobility time (R: 55.429 ± 10.064 [mean ± SD]; NR: 78.896 ± 2.186; *p* = 0.004). These results indicated that ESC could reverse the depressive-like behavior in R group not in NR group.

### Dynamic changes of the gut microbial diversity

We explored the dynamic changes of the gut microbiome in depressive mice. A total of 4 samples were excluded from the analysis due to insufficient sample volumes. Totally, 151 fecal samples were collected prior to ESC administration (day 0) and at 4 weeks post administration (end of study). A total of 7155,561 high-quality 16 S rRNA sequences were generated with an average of 47,387 reads per sample. After rarefaction of sample to an equal sequencing depth (28,216 reads per sample) and clustering, 4260,616 sequences from 151 fecal samples were grouped into 1289 OTUs for the downstream analysis.

**Fig. 2:**
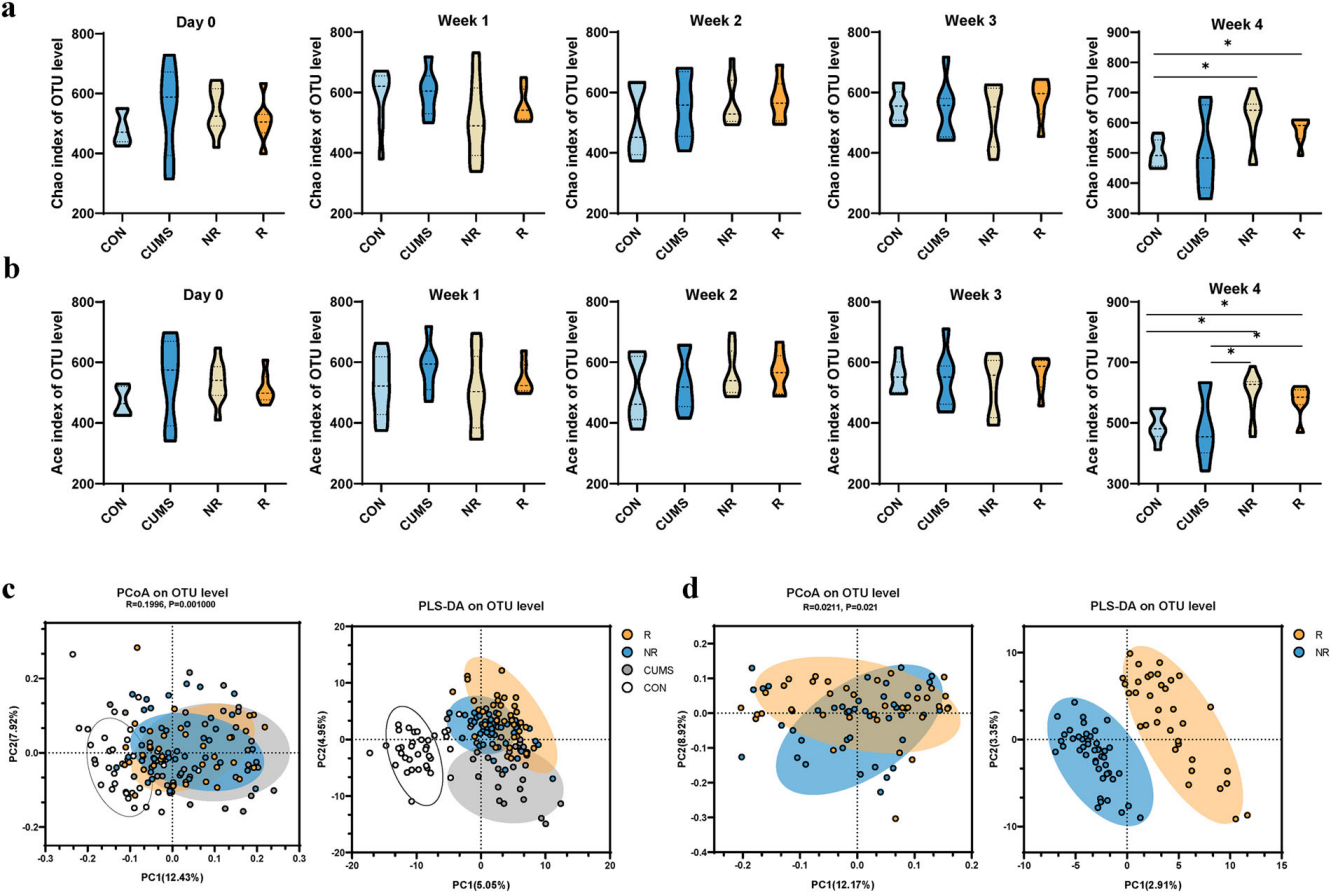
Longitudinal changes in microbial diversity in different groups. Bacterial richness, here presented by Chao indices (**a**) and Ace indices (**b**) obtained at different time points from day 0 to week 4, NR and R group was higher than those in CON and CUMS groups in week 4. Data are presented with means and standard errors (*p < 0.05, Wilcoxon rank-sum test). Principal coordinate analysis (PCoA) plots based on unweighted UniFrac distances and the partial least squares-discriminant analysis (PLS-DA) showed distinct clusters on the OTU level among the four groups (**c**), and between the R and NR group (**d**), the bacterial communities of the R and NR group clustered separately. Statistical significance was determined by an ANOSIM with 999 permutations. The percentage of variation explained by principal co-ordinates is marked on the axes.

We have investigated the effect of ESC on microbial diversity, and found that microbial richness including the Chao and Ace index are higher in the NR and R group at week 4, compare to the controls (all *p* < 0.05) (Fig. 2a, b). For Ace index only, NR and R group was higher than those in CON and CUMS groups (all *p* < 0.05) (Fig. 2b). No significant changes in community richness during the first 3 weeks and in community diversity during the entire treatment were observed (Fig. 2, Fig. S1).

To evaluate the changes of β-diversity of gut microbiota across different groups, principal coordinate analysis (PCoA) based on the unweighted Unifrac distance matrixes and partial least squares-discriminant analysis (PLS-DA) were conducted (Fig. 2c, d, Fig. S2). Consequently, we found that the four groups were clustered into distinct groups with beta-diversity estimates, as assessed by the ANOSIM tests (R = 0.1996, *p* = 0.001, Fig. 2c). Especially, microbial composition of R group was significantly different from that in the NR group (R = 0.0211, *p* = 0.021, Fig. 2d).

Moreover, the dynamic changes of β-diversity was also conducted, during the first 3 weeks control group was well separated from other groups (ANOSIM, all *p* < 0.01) (Fig. S2a), but the other three groups were more similar to each other. However, at week 4, a discriminative trend was observed between R and NR groups based on PCoA (unweighted Unifrac distance) (Fig. S2b), while PLS-DA plots showed a more clearly discrimination between the two groups (Fig. S2c).

### Dynamic changes of the gut microbiome in CUMS group compare to CON group

We first analyzed the alteration of the gut microbiota at last 4 weeks of CUMS procedure. The relative abundance of *Firmicutes* was decreased in CUMS group, while *Bacteroidetes* was increased, the most pronounced differences were observed at week 4 (*p* < 0.01, Figs. S3a, S4a). *Camplibobacterota* was constantly higher in the CUMS group than the CON group, while *Actinobacteriota* was lower in the CUMS group. At the family level, we found that the relative abundance of *Bacteroidaceae*, *Helicobacteraceae,* and *Rikenellaceae* were higher in CUMS group than CON group for all time points, while *Eggerthellaceae* and *Bifidobacteriaceae* showed a lower level in CUMS group (Figs. S3b, S4b). Similar to family level, consistent alterations of *Bacteriodes* and *Bifidobacterium* was observed at genus level (Figs. S3c, S4c). These data showed significant variations of gut microbiota compositions between CUMS and CON groups, which indicated that perturbations of intestinal homeostasis could be induced by CUMS procedure.

### The differences in microbial composition between R and NR group

**Fig. 3:**
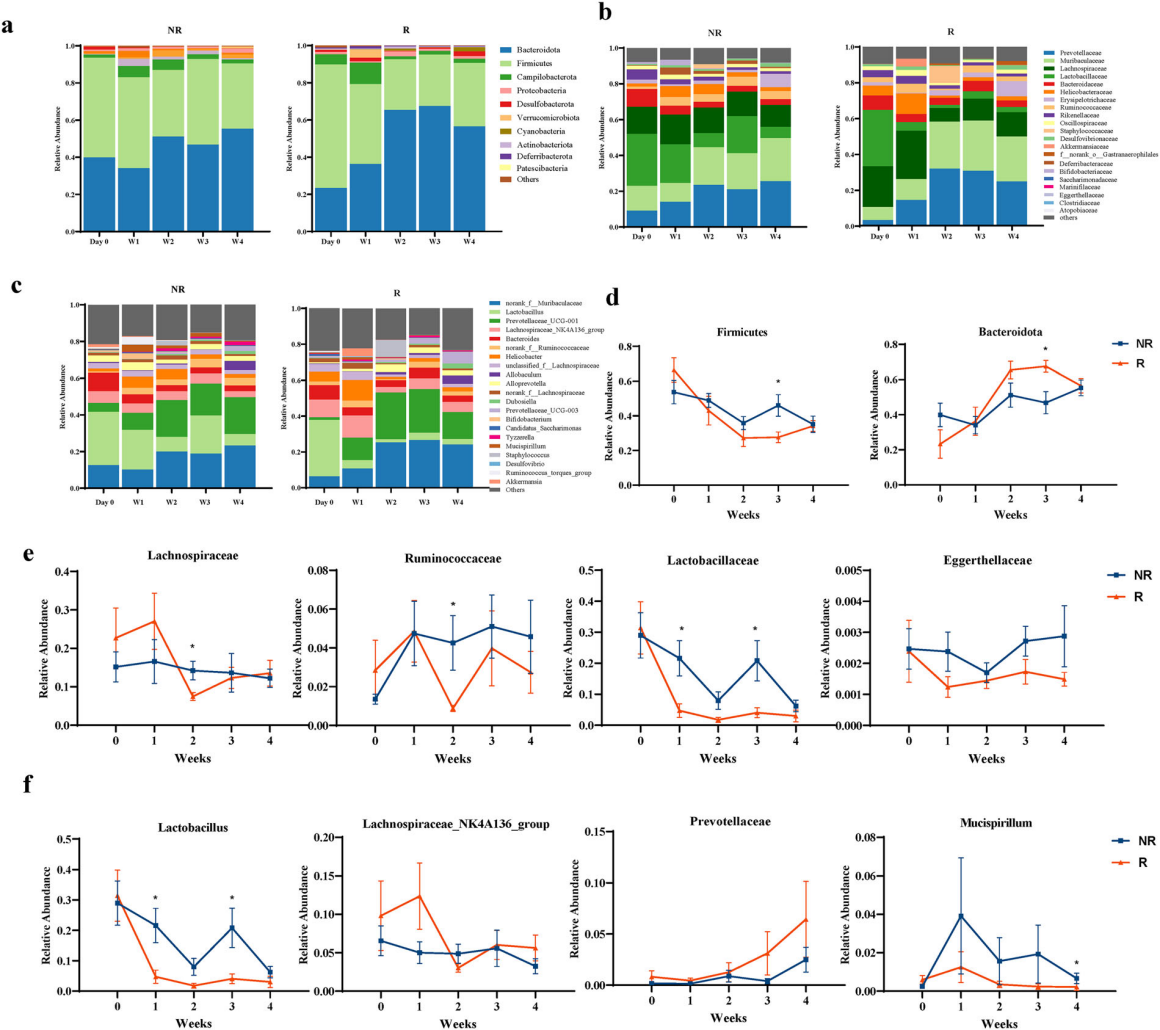
Longitudinal changes in microbial composition between R and NR group. Stacked bar chart showing the longitudinal changes of the gut microbial composition in mice at the phylum (**a**), family (**b**) and genus (**c**) level between the two groups. Plots show the relative abundance of dominant bacterial phylum (**d**), family (**e**) and genus (**f**) at different stages. (*p < 0.05, Wilcoxon rank-sum test).

Given that the depressive mice showed divergent response to ESC treatment, it is interesting to investigate the association between the gut microbiome and the responses to ESC. Thus, we have investigated the dynamic changes in microbial composition between R and NR groups. Before treatment initiation, *Firmicutes* and *Bacteroidetes* dominated the gut microbiota of both R and NR. Specifically, *Bacteroidetes* were most abundant, followed by *Firmicutes* and *Campilobacterota* (Fig. 3a, d). The family *Lachnospiraceae* and *Ruminococcaceae* was found to be significantly more abundant (*p* < 0.05) in NR group at 2 weeks of escitalopram administration. *Lactobacillaceae* and *Eggerthellaceae* showed lower levels in R group after treatment initiation until 4 weeks (Fig. 3b, e). At genus level, *Lactobacillus* were most abundant in NR group, and the obvious difference appeared at week 1 and week 3 (*p* < 0.05). In addition, *Prevotellaceae_UCG-003* showed an increasing trend in R group, while remain stable at a low level in NR group (Fig. 3c, f). No difference was noted in other dominant taxa between R and NR group (Fig. S5).

### Differential OTUs associated with the response to escitalopram

**Fig. 4:**
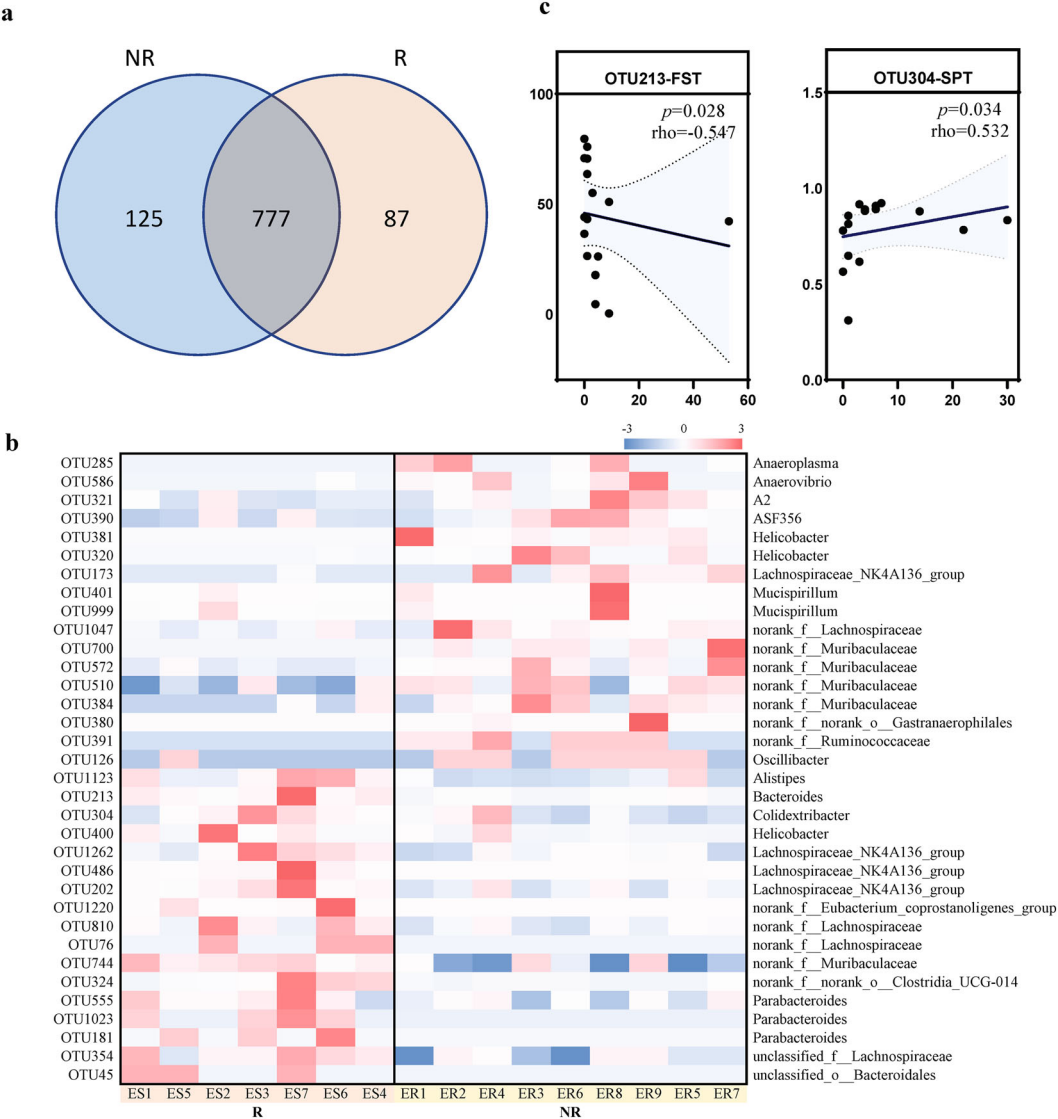
Analysis of the OTUs enriched in R and NR groups in week 4. **a** Venn diagram showing the distribution of the OTUs between R and NR groups, 777 OTUs were shared between R and NR groups. **b** Heat map of discriminant OTUs determined by LEfSe analysis in fecal samples between R and NR group with the double criteria of both LDA > 2 and *p* < 0.05. Each column represents an individual sample, OTUs are classified in genus format on the right side. **c** Scatter diagram of the relative abundances of the behavior-related OTUs. The correlation was tested by Spearman correlation analysis. FST, forced swim test. SPT, sucrose preference test.

In order to obtain deeper insight of microbiota alterations upon ESC administration, linear discriminant analysis effect size (LEfSe) was performed to compare microbial composition at 4 weeks of the 16 S rDNA operational taxonomic unit (OTU) level. As shown in the Venn diagram in Fig. 4a, 777 OTUs were shared between R and NR groups, while 125 were unique to NR and 87 to R. A total of 34 OTUs that significantly differed between R and NR groups (*p* < 0.05; linear discriminant analysis [LDA] > 2.0) were shown in Fig. 4b, of which 17 OTUs were enriched in R group and 17 OTUs in NR group. Among those differential OTUs, 3 OTUs (OTU1262, OTU486, OTU202) belong to genus *Lachnospiraceae_NK4A136*, and 3 OTUs (OTU555, OTU1023, OTU181) belong to genus *Parabacteroides* were enriched in R group. 4 OTUs (OTU700, OTU572, OTU510, OTU384) belong to family *Muribaculaceae*, 2 OTUs (OTU381, OTU320) belong to genus *Helicobacter* and 2 OTUs (OTU401, OTU999) belong to genus *Mucispirillum* were enriched in NR group. To further investigate whether the differential OTUs were associated with depressive-like symptoms, spearman rank correlation analysis was performed of OTUs and behavior indices between the R and NR groups. OTU213 (belong to genus *Bacteroides*) showed negative correlation with immobility time in FST (rho = −0.547, *p* = 0.028), and OTU304 (belong to genus *Colidextribacter*) showed positive correlation with sucrose intake in SPT (rho = 0.532, *p* = 0.034) (Fig. 4c).

### Disturbances of serum metabolic signatures between R and NR group

**Fig. 5:**
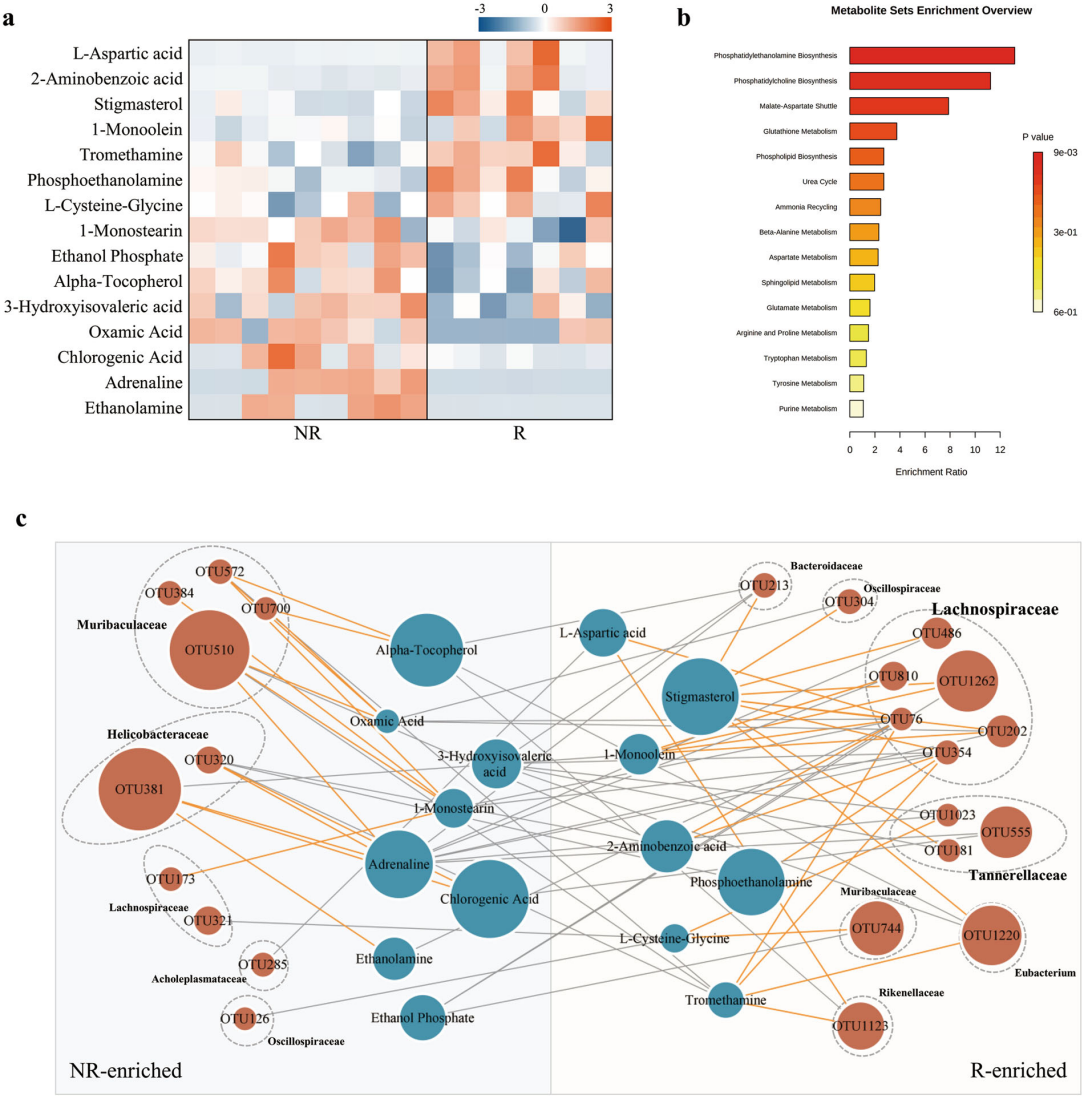
Metabolomic analysis for serum samples in R and NR groups. **a** Heat map based on differentially abundant metabolites between R and NR groups determined by LEfSe analysis LDA > 2 and *p* < 0.05, 7 and 8 metabolites were enriched in R and NR group, respectively. **b** Pathway enrichment analysis of differential abundant metabolites, the phosphatidylethanolamine biosynthesis is the predominant metabolic pathway. **c** Interaction networks among significant OTUs and metabolites, size of node indicates abundance, different colors of nodes represent metabolites (blue) and OTUs (orange), negative correlations are colored in lines of grey and positive correlations are shown in lines of orange, thickness of the lines reflects the strength of the correlation (Spearman correlations with rho > 0.5, *p* < 0.05).

Given gut microbiome often modulates the host’s metabolic pathways, here GC-MS based metabolomics was used to compare the metabolic characteristics of R and NR groups. Totally, 15 metabolites were found to be the differential metabolites between R and NR group (VIP > 0 1, *P* < 0.05), as shown in Fig. 5a. Specifically, R-enriched metabolites included L-aspartic acid, 2-aminobenzoic acid, stigmasterol, 1-monoolein, tromethamine, phosphoethanolamine, L-cysteine-glycine. While, the NR-enriched metabolites were composed of 1-monostearin, ethanol phosphate, alpha-tocopherol, 3-hydroxyisovaleric acid, oxamic acid, chlorogenic acid, adrenaline, and ethanolamine. These differential metabolites were mainly involved in phospholipid metabolism (phosphatidylethanolamine and phosphatidylcholine biosynthesis) (Fig. 5b). Together, the serum metabolomic analyses showed that the divergent respond to ESC was characterized by disturbances of phospholipid metabolism.

### Co-occurrence network analysis between the gut microbiota and serum metabolites in R and NR group

To investigate the potential reciprocal interactions between altered gut bacteria and serum metabolites, a co-occurrence network was constructed based on Spearman correlation analysis. We found that 6 OTUs (OTU486, OTU810, OTU1262, OTU76, OTU354, OTU202) that belong to *Lachnospiraceae* formed a strong co-occurring relationship with serum metabolites assigned to stigmasterol. 2 OTUs (OTU76 belong to *Lachnospiraceae*, OTU1123 belong to *Rikenellaceae*) were positively correlated with L-aspartic acid (Fig. 5c). Notably, there are 2 OTUs belong to *Helicobacteraceae* (OTU381, OTU510, OTU320) and 4 OTUs belong to *Muribaculaceae* (OTU384, OTU572, OTU700, OTU510) formed a strong co-occurring relationship with NR-enriched serum metabolites. Moreover, 7 OTUs (OTU354, OTU1023, OTU202, OTU213, OTU486, OTU555, OTU810) that enriched in R groups were negatively correlated with adrenaline (Fig. 5c). These findings indicate that altered gut microbiota and metabolites formed a synergistic and node-related co-occurrence network between R and NR group.

## Discussion

In present study, we have investigated the dynamic changes of gut microbiota and serum metabolites in CUMS mice model of depression treated with ESC based on 16 S rRNA sequencing and metabolomics. We found that the ESC responder and non-responder groups were characterized by differential microbial composition and metabolic pathways. R group were characterized by enriched genus *Prevotellaceae_UCG-003*, and depleted families *Lactobacillaceae* and *Eggerthellaceae*. Moreover, bacterial OTUs belonged to families *Lachnospiraceae*, *Muribaculaceae*, and *Helicobacteraceae* formed strong co-occurring relationships with several serum metabolites assigned to phospholipid metabolism. Our results indicated that the gut microbiome might be involved in modulating the clinical response to ESC therapy, which might be mediated through different trajectories of microbiome shifts and the interaction between microbiota and metabolites.

In present study, ESC was shown to reduce the depressive symptoms (increased sucrose preference ratio in SPT and decreased immobility time in FST) of CUMS mice in responder group, while not in non-responder group. Based on 16 S rRNA sequencing, a high microbial richness (Chao and Ace index) at week 4 and an increasing trend over the entire treatment were observed in NR and R group compare to the controls. These findings suggested that ESC would attribute to the elevated alpha-diversity of gut microbiome. Previous studies have found that several commonly used non-antibiotic drugs, including proton pump inhibitors (PPIs) and metformin, could change the composition and function of gut microbiome, while antidepressants such as SSRIs and TCAs showed no effect on alpha-diversity of gut microbiome^15,29^. A recent study showed that five antidepressants, except desipramine, reduced the richness of microbial communities, but did not affect their evenness^30^, this finding was inconsistent with our results which may due to the different drugs and treatment duration, which need more validation. The present study showed that bacterial diversity was significantly different across all four groups, especially R group was clearly distinguished from NR groups. Consistent with our prior studies, the gut microbial composition of MDD subjects differed from that in the healthy controls based on unweighted Unifrac distance^14^. Moreover, the difference in the composition of gut microbiome in patients related to the therapeutic efficacy of anti-PD-1 monoclonal antibody was reported^31^. In addition, we have investigated the longitudinal changes of beta-diversity among the four groups. During the first 3 weeks, only control group was well separated from other groups, while at week 4 a discriminative trend was observed among CUMS, R and NR groups, which indicated that the beta-diversity was dynamic changes with the treatment period. In addition, a more clearly discrimination between R and NR groups was observed at week 4.

The dynamic alternations of microbial composition were also analyzed, and our data showed differential patterns of succession of the mice gut microbiome at different time points induced by CUMS procedure. Dominant bacterial family *Firmicutes* appeared to have higher abundance in CON group compared to CUMS group after week 1, while *Bacteroidetes* showed an opposite trend, which was consistent with our previous research that *Bacteroidetes* were higher in MDD patients relative to HC^10^. *Firmicutes* are reported to have ability to protect against intestinal barrier dysfunction through fermenting the carbohydrates into various short-chain fatty acids, and healthy individuals appeared to have higher abundance of this phylum^32,33^. The disturbances of microbial composition in CUMS mice are in agreement with precious reports that stress resulted in microbial dysregulation^34^.

Above findings indicated that the CUMS procedure affects the gut microbial structure, while ESC administration results in divergent responds in depressive mice, which lead us to explore whether the distinct shifts in microbial structure associated with the ESC efficiency? Therefore, we have investigated the alteration of bacterial taxa during different stages of ESC administration between R and NR groups. At the phylum level, differences in *Firmicutes* and *Bacteroidota* abundance were found between R and NR groups at week 3 and disappear at week 4. It is possible that divergent microbial succession patterns of sub-levels of bacterial taxa such as the alterations of several genus or species. Family *Ruminococcaceae* was found to be significantly more abundant in NR group at 2 weeks of ESC administration, and remain steadily through time. Interestingly, we have found that *Lactobacillaceae* showed lower levels in R group after treatment initiation until 4 weeks, meanwhile the genus *Lactobacillus* belonging to this family showed a similar pattern of development. *Lactobacillus* is generally considered to be a beneficial participant, and could prevent inflammation and protect the gut against multiple pathogens and bacteria. However, recent findings on *Lactobacillus* in both human and animal researches also mentioned that it may be associated with several diseases^35,36^. Thus, the impact of *Lactobacillus* on drug efficiency deserves further research. The levels of *Prevotellaceae_UCG-003* were higher in R group in comparison with NR group, but the difference was not significant. The higher levels of *Prevotellaceae_UCG-003* might have the potential to modulate intestinal inflammation by producing succinate to activates dendritic cells^37^. Hence, *Prevotellaceae_UCG-003* might play positive roles in immune-inflammatory system, consequently influencing the ESC efficacy and alleviating depressive-like behaviors in mice.

To deeply investigate whether there was specific microbial cluster that was difference between R and NR groups, we then compared the microbial composition at the OTU level. This analysis revealed that 125 OTUs were uniquely present in NR group, 87 OTUs were uniquely present in R group and 777 OTUs were shared between R and NR groups. Based on LEfSe algorithm analysis, we found 17 R-enriched OTUs and 17 NR-enriched OTUs. Among the R-enriched OTUs, 3 OTUs belonged to genus *Lachnospiraceae_NK4A136*, and 3 OTUs belonged to genus *Parabacteroides*. *Lachnospiraceae* is considered to be beneficial to the host health via the production of anti‐inflammatory SCFAs and helps to maintain the integrity of the intestinal barrier^38^. *Parabacteroides* has been shown to provided protective benefits in neurologic disorders through altering the levels of neurotransmitters in the brain, including glutamate and GABA in the hippocampus^39^. On the contrary, 2 OTUs (OTU381, OTU320) belong to genus *Helicobacter* were enriched in NR group. *Helicobacter* was reported to stimulate the responses of pro‐inflammatory cytokines such as Th1 and Th17 in immunocompromised mice^40^. Previous study showed that inflammation may contribute to nonresponse to antidepressants^41^. Among the R-enriched OTUs, OTU304 (belong to genus *Colidextribacter*) was positively correlated with the anti-depressive-like behavior (sucrose intake in SPT), which is required to investigate in further studies. Together, our data have demonstrated that the specific gut microbial taxa may be a hallmark of response to antidepressant treatment.

Previous experiments have found that depression is characterized by disturbances of several metabolites, but the metabolic changes involved in antidepressant treatment were not studied yet. Here, we observed that the disturbances of serum metabolomics between R and NR groups. Serum metabolite profiling showed that 15 metabolites were differentially expressed between R and NR groups. These differential metabolites were mainly involved in phosphatidylethanolamine and phosphatidylcholine biosynthesis. Co-expression network showed that the alternations of bacterial OTUs were substantially correlated with the differential metabolites. We found that 5 R-enriched OTUs which belong to family *Lachnospiraceae*, were positively correlated with stigmasterol. The antidepressant effects of stigmasterol, and the effects on neurotransmitter systems and mechanisms involved in antidepressant effects have been observed^42^. Stigmasterol was found to be resistant to bacterial saturation or degradation, however, the metabolic mechanisms associated with gut microbiota are still poorly understood. Interestingly, we also found that 7 R-enriched OTUs were negatively correlated with adrenaline, and 3 NR-enriched OTUs were positively correlated with adrenaline. Escitalopram as a SSRI has very low affinity with adrenergic receptors. The high levels of adrenaline in NR group might due to the overactive of the hypothalamic-pituitary-adrenal axis or the result of compensatory modifications of adrenaline^43^. These findings suggest that disturbances of the gut microbiome may affect the occurrence of different response to ESC through shared metabolic pathways. In future studies, the interaction between these metabolites involving in phospholipid metabolism and gut microbiota should be deeply explored, to clarify their underlying roles in the efficiency of antidepressant drugs.

It should be mentioned that there are some limitations in this study: (i) As with all experimental models, limitations of using CUMS mice model of depression to recapitulate the pathophysiology of human depression must be considered, and further studies are needed before mouse data can be translated into human studies; (ii) Due to limited sample sizes, metabolomics analysis was conducted only in R and NR groups; (iii) the roles of serum metabolites associated with the differential gut microbes in the antidepressant response require further validation.

Taken together, we illustrated that the dynamic-variation characteristics of the gut microbiome and the relationship with metabolites in CUMS mice with divergent escitalopram responses. The different trajectories of microbiome shift between drug responder and non-responder groups were observed. The presence of several bacterial taxa such as NR-enriched families *Ruminococcaceae* and *Lactobacillaceae* and R-enriched genus *Prevotellaceae_UCG-003*, which might be used for predicting antidepressant treatment. Furthermore, our results revealed R-enriched OTUs belonging to *Lachnospiraceae* were positively correlated with stigmasterol, which indicated that the bi-directional influence might be occurred between the gut microbiome and metabolome, may affect the anti-depressive therapeutic effect. Our study shows the potential of gut microbiota as a therapeutic target, and the related bacterial taxa and metabolic changes can be used as a regulatory strategy to provide better therapeutic options for depression.

## Supplementary information

Supplementary Figure legends

FigureS1

FigureS2

FigureS3

FigureS4

FigureS5
