# Supplementary material for: Characterization of gut microbiome in mice model of depression with divergent response to escitalopram treatment
Source: Transl Psychiatry. 2021 May 20;11:303. doi: 10.1038/s41398-021-01428-1 (PMC8138009; doi:10.1038/s41398-021-01428-1)
Supplement: Supplementary file 1 — Supplementary Figure legends [file 41398_2021_1428_MOESM1_ESM.docx]

**Supplementary Figure legends**

**Fig.S1 Longitudinal changes in microbial alpha-diversity in different groups**. Community diversity, here presented by Shannon indices**(a)** and Simpson indices**(b)** obtained at different time points from day0 to week4. Data are presented with means and standard.

**Fig.S2 Longitudinal changes in microbial β-diversity in different groups**. **(a)** PCoA plots based on unweighted UniFrac distances between samples from Day0 to Week3. **(b)** Principal coordinate analysis (PCoA) plots based on unweighted UniFrac distances and **(c)** the partial least squares-discriminant analysis (PLS-DA) showed distinct clusters on the OTU level among the four groups in week4. The percentage of variation explained by principal coordinates is marked on the axes.

**Fig.S3 Longitudinal changes in microbial composition in different groups.** Stacked bar chart showing the longitudinal changes of the gut microbial composition in mice at the phylum **(a)**, family **(b)** and genus **(c)** level between CON and CUMS groups.

**Fig.S4 Gut microbiota development through time between CON and CUMS group**. Plots show the relative abundance of dominant bacterial phylum**(a)**, family**(b)** and genus**(c)** at different stages. (*p＜0.05, Wilcoxon rank-sum test)

**Fig.S5 Gut microbiota development through time between R and NR group**. Plots show the relative abundance of dominant bacterial phylum**(a)**, family**(b)** and genus**(c)** at different stages. (*p＜0.05, Wilcoxon rank-sum test)
