# Supplementary figures and images for: Characterization of gut microbiome in mice model of depression with divergent response to escitalopram treatment
Source: Transl Psychiatry. 2021 May 20;11:303. doi: 10.1038/s41398-021-01428-1 (PMC8138009; doi:10.1038/s41398-021-01428-1)

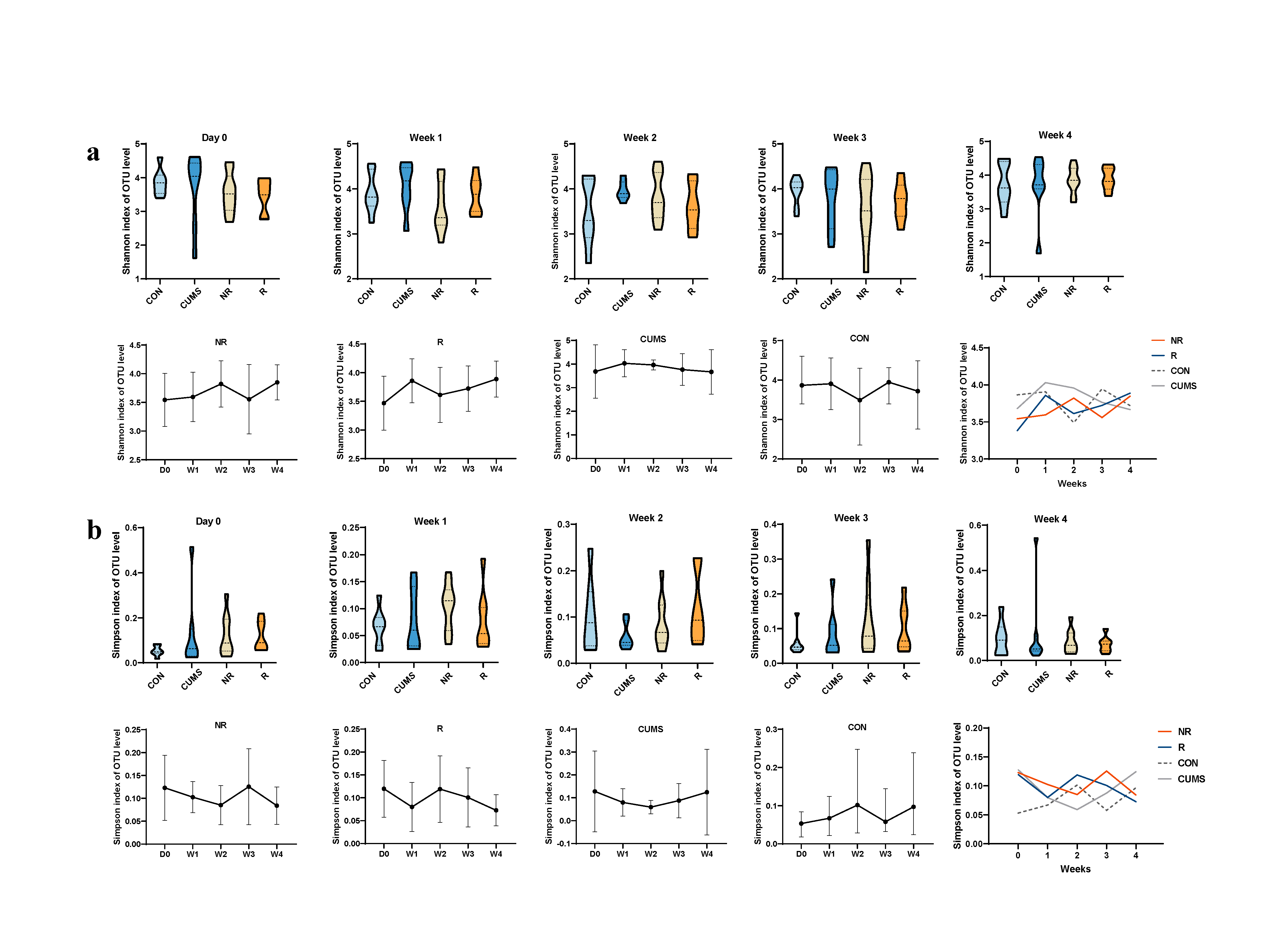

Supplement: Supplementary file 2 — FigureS1 [file 41398_2021_1428_MOESM2_ESM.tif]

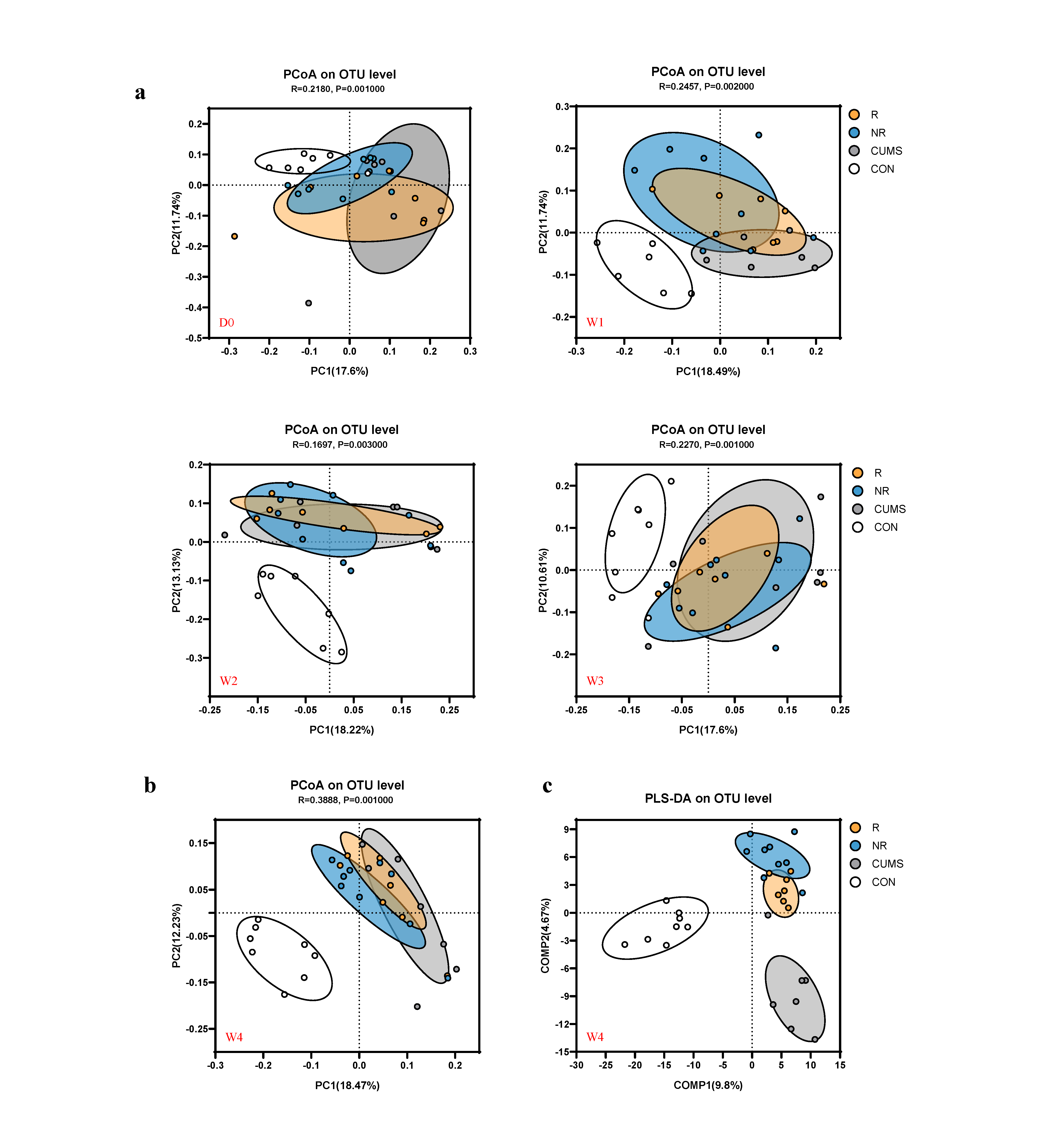

Supplement: Supplementary file 3 — FigureS2 [file 41398_2021_1428_MOESM3_ESM.tif]

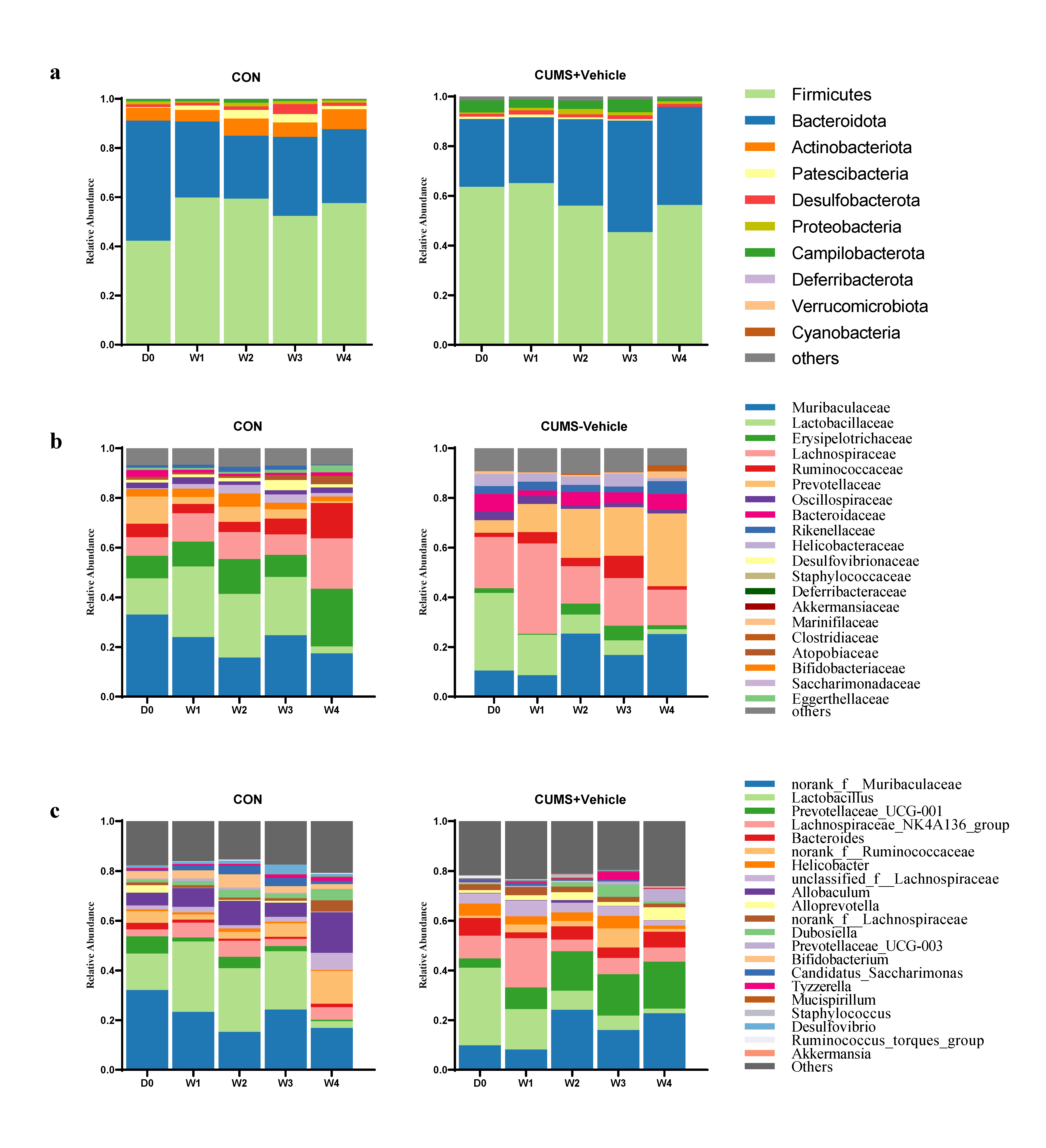

Supplement: Supplementary file 4 — FigureS3 [file 41398_2021_1428_MOESM4_ESM.tif]

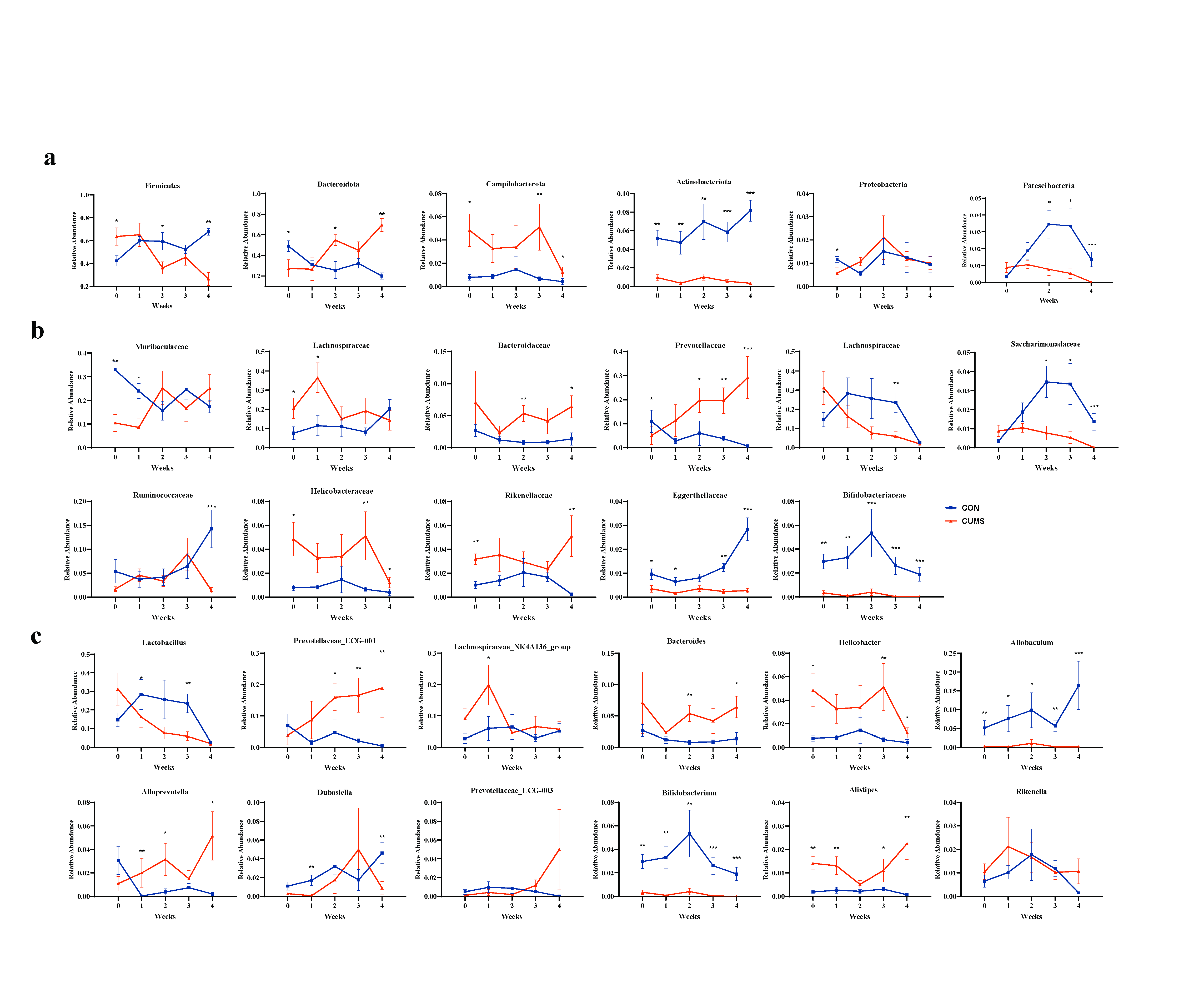

Supplement: Supplementary file 5 — FigureS4 [file 41398_2021_1428_MOESM5_ESM.tif]

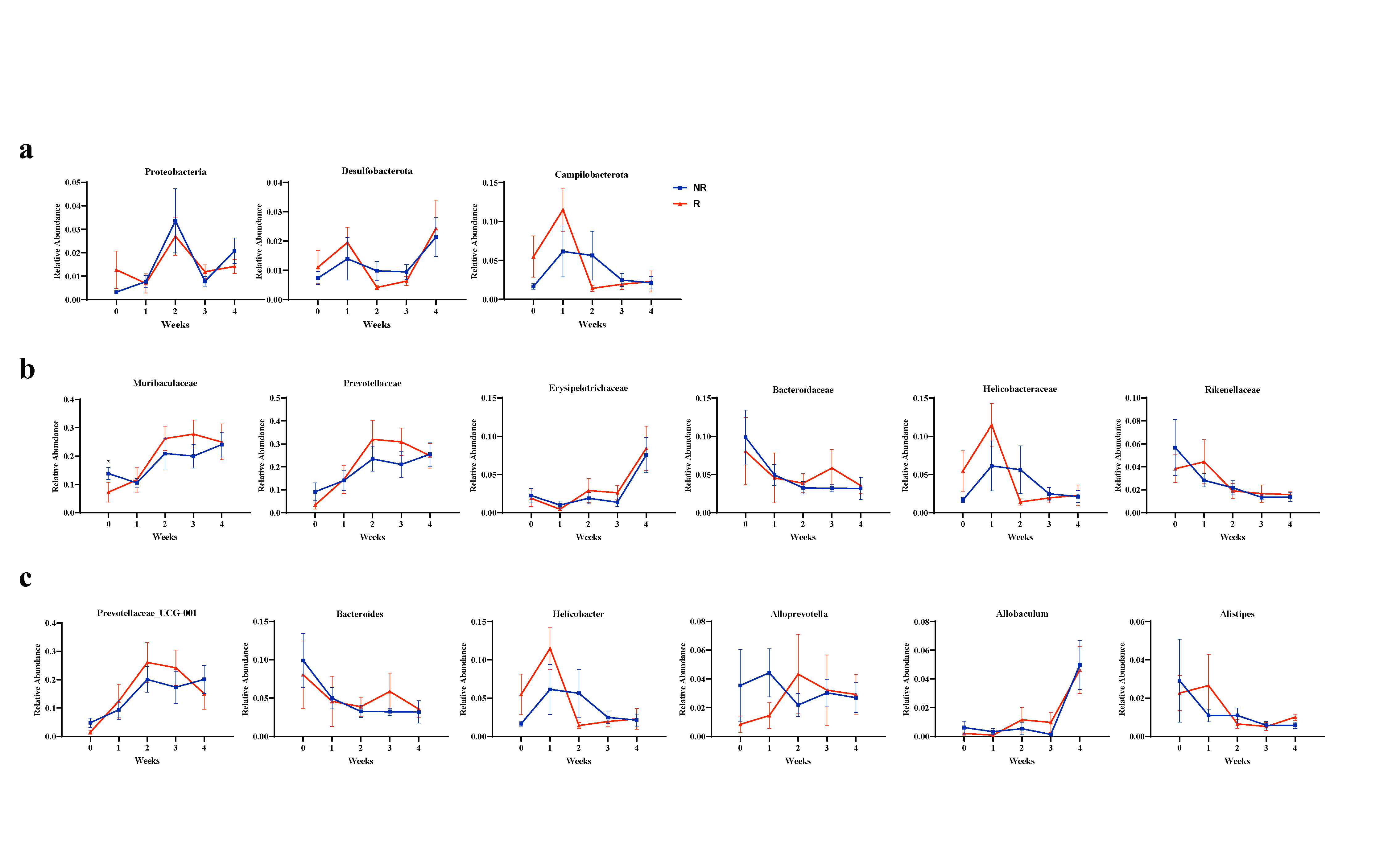

Supplement: Supplementary file 6 — FigureS5 [file 41398_2021_1428_MOESM6_ESM.tif]
